# Integrated Analysis of microRNA and mRNA Transcriptome Reveals the Molecular Mechanism of *Solanum lycopersicum* Response to *Bemisia tabaci* and *Tomato chlorosis virus*

**DOI:** 10.3389/fmicb.2021.693574

**Published:** 2021-06-22

**Authors:** Hao Yue, Li-Ping Huang, Ding-Yi-Hui Lu, Zhan-Hong Zhang, Zhuo Zhang, De-Yong Zhang, Li-Min Zheng, Yang Gao, Xin-Qiu Tan, Xu-Guo Zhou, Xiao-Bin Shi, Yong Liu

**Affiliations:** ^1^Subcollege of Longping, Graduate School of Hunan University, Changsha, China; ^2^Hunan Academy of Agricultural Sciences, Institute of Plant Protection, Changsha, China; ^3^Institute of Vegetable, Hunan Academy of Agricultural Sciences, Changsha, China; ^4^Department of Entomology, University of Kentucky, Lexington, KY, United States

**Keywords:** *Solanum lycopersicum*, *Tomato chlorosis virus*, Bemisia tabaci, Transcriptome, MicroRNA

## Abstract

*Tomato chlorosis virus* (ToCV), is one of the most devastating cultivated tomato viruses, seriously threatened the growth of crops worldwide. As the vector of ToCV, the whitefly *Bemisia tabaci* Mediterranean (MED) is mainly responsible for the rapid spread of ToCV. The current understanding of tomato plant responses to this virus and *B. tabaci* is very limited. To understand the molecular mechanism of the interaction between tomato, ToCV and *B. tabaci*, we adopted a next-generation sequencing approach to decipher miRNAs and mRNAs that are differentially expressed under the infection of *B. tabaci* and ToCV in tomato plants. Our data revealed that 6199 mRNAs were significantly regulated, and the differentially expressed genes were most significantly associated with the plant-pathogen interaction, the MAPK signaling pathway, the glyoxylate, and the carbon fixation in photosynthetic organisms and photosynthesis related proteins. Concomitantly, 242 differentially expressed miRNAs were detected, including novel putative miRNAs. Sly-miR159, sly-miR9471b-3p, and sly-miR162 were the most expressed miRNAs in each sample compare to control group. Moreover, we compared the similarities and differences of gene expression in tomato plant caused by infection or co-infection of *B. tabaci* and ToCV. Taken together, the analysis reported in this article lays a solid foundation for further research on the interaction between tomato, ToCV and *B. tabaci*, and provide evidence for the identification of potential key genes that influences virus transmission in tomato plants.

## Introduction

Plant viruses seriously threaten the growth and development of crops, which has become a worldwide problem (Doughari, 2015; Peixoto et al., 2017). The majority of plant viruses must be transmitted through specific vectors to ensure their survival (Moreno et al., 2012; Whitfield et al., 2015). Complex interactions between host plant, plant viruses and their insect vectors are an essential molecular interface that determines acquisition from infected host plants and transmission to new hosts (Dietzgen et al., 2016; Carr et al., 2018). Recently, more and more studies have focused on the interaction of plants, viruses, and insects to understand the molecular mechanisms among them (Şahin-Çevik et al., 2019).

*Tomato chlorosis virus* (ToCV) (genus *Crinivirus*, family *Closteroviridae*), was first identified in Florida of the Unite States and seriously threatened tomato growth in worldwide (Wisler G. C. et al., 1998; Wisler G. et al., 1998; Orfanidou et al., 2014; Lee et al., 2018); the virus can reduce tomato yield more than 50% (Lozano et al., 2006; Velasco et al., 2008), and its latent period can be up to 30 days (Favara et al., 2019; Fiallo-Olivé and Navas-Castillo, 2019). ToCV is a phloem virus and it is mainly transmitted by the vector whitefly in a semi-persistent manner (Wisler G. C. et al., 1998; Zhao et al., 2013; Polston et al., 2014). In China, ToCV has reached an outbreak level in several areas, including Beijing, Tianjin, Shandong, Hunan, Jiangsu, and Guangdong (Zhao et al., 2013; Liu et al., 2014; Wang et al., 2018; Wei et al., 2018), and still expanding its geographical and host ranges (Fiallo-Olivé and Navas-Castillo, 2019).

The whitefly, *Bemisia tabaci* (Hemiptera: Aleyrodidae), consisting of at least 39 cryptic species (Alemandri et al., 2015), as an important vector of ToCV, causes severe damage to crops worldwide (Xia et al., 2019). The previous study showed that *B. tabaci* can acquire ToCV in 24 h after feeding of ToCV-infected tomato plants (Ding et al., 2019). Among all the known biotypes of whitefly, MED and MEAM1 are the most invasive, and they are widely distributed around the world, causing serious damage (De Barro et al., 2011). The whitefly MED was first detected in China in 2003, and has gradually displaced MEAM1 and become the dominant cryptic species of *B. tabaci* (Chu et al., 2006; Pan et al., 2011). Studies revealed that ToCV was retained in *B. tabaci* MED adults more than 4 days, and MED had higher transmission efficiency than MEAM1 (Polston et al., 2014; Shi et al., 2018). There are still no ToCV-resistant varieties on the market, and *B. tabaci* has been reported to be resistant to many chemicals (Cui et al., 2019), which increases the difficulty of controlling ToCV.

With the development of next-generation sequencing technology, research on the interaction between plants, insect vectors, and viruses has made rapidly progress (Nouri et al., 2018). Transcriptome analysis revealed that defense-related pathways and oxidative stress in wheat were rapidly induced within hours after the initiation of aphid feeding, and that nicotinamide adenine dinucleotide phosphate (NADPH) oxidase plays an important role in aphid-induced defense responses and hydrogen peroxide (H_2_O_2)_ accumulation in wheat (Zhang et al.,2020a,b). Many differentially expressed genes (DEGs) involved in photosynthesis, plant-pathogen interactions, secondary metabolism, and plant hormone signal transduction in watermelon response to *Cucumber green mottle mosaic virus* infection (Sun et al., 2019). Evidence revealed that miRNAs involved in immunity to various pathogens, including bacteria, fungus, virus and parasites (Pérez-Quintero et al., 2010; Feng H. et al., 2014; Zhao et al., 2015; Liu et al., 2019). For example, the accumulation level of miR159ab was correctly associated with severity of viral disease symptoms (Du et al., 2014). Expression levels of the gibberellins (GA) biosynthesis genes were drastically reduced in Tomato planta macho viroid and Mexican papita viroid infected tomato plants (Aviña-Padilla et al., 2018). Potato virus Y (PVY)-infected tomato plants could regulate expression pattern of miRNA pathways several defense responses mediated by nucleotide-binding leucine-rich repeat proteins (NLRs) and receptor-like protein (RLPs)/receptor-like kinases (RLK) receptors (Prigigallo et al., 2019). Early studies also revealed that ToCV induced the basic defense response and activated the defense signaling in tomato plants (Şahin-Çevik et al., 2019). However, there is little report about the molecular mechanism of tomato plant caused by infection or co-infection of *B. tabaci* and ToCV.

In our results, the accumulation of virus increases most rapidly at 40 days. Moreover, consistent with previous study (Shi et al., 2014), the content of jasmonic acid (JA), salicylic acid (SA) and antioxidant enzyme activities in tomato plants were changed significantly after feeding of *B. tabaci* adults (virus-free) for 24 h. To further investigate the molecular mechanism of tomato plant caused by infection or co-infection of *B. tabaci* and ToCV, this work adopts transcriptome techniques of microRNA and mRNA analysis in tomato leaves under interaction with *B. tabaci* MED and ToCV to (1) explore the molecular mechanism of tomato plants’ responses; (2) provide evidence for comparison of differential gene expression affected by ToCV and its vector *B. tabaci* MED; (3) establish a fundamental understanding on interactions between ToCV, *B. tabaci* MED and tomato plants. In addition, we compared the similarities and differences of gene expression between tomato plants feeding by *B. tabaci* MED and ToCV-infected tomato plants. The results provide a comprehensive view of the molecular response to *B. tabaci* MED and ToCV in tomato plants and yield new insights into the interactions between insect vectors and multiple viruses.

## Results

### Accumulation of ToCV in Tomato Plants, Analysis of Plant Endogenous Hormone and Antioxidant Enzyme Activities

**FIGURE 1:**
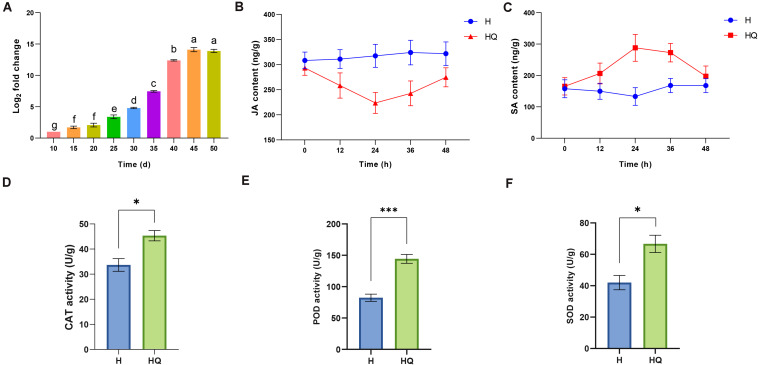
Determination of *tomato chlorosis virus* (ToCV) content, plant hormones and antioxidant enzyme activity. **(A)** Accumulation of *tomato chlorosis virus* in tomato plants at different time points. **(B,C)** The JA and SA content in control and treatment group. **(D–F)** The content of CAT, PDO, and SOD in control and treatment group. Values are means ± SE. For each time, means with different letters and asterisk are significantly different (*P* < 0.05). H, healthy tomato plants. HQ, tomato plants was fed on by healthy *B. tabaci* for 24 h. V, tomato plants was inoculated with ToCV. VQ, tomato plants with ToCV that were fed on by healthy *B. tabaci* for 24 h.

RT-qPCR was used to detect and quantify ToCV accumulation in tomato plants at different time points from 10 to 50 days. As shown in Figure 1A, the viral titer grew continuously from 10 to 45 days and showed a rapid increase from 30 to 40 days, reaching a peak at 45 days. After 45 days, viral titer remained at a relatively stable level. Notably, we found the accumulation of virus increases most rapidly at 40 days from our data. This may mean the interaction between plants and viruses is the most intense and complex at that time point. Therefore, in order to obtain more molecular mechanisms of interaction between tomato plants and ToCV, samples from each treatment at 40 days were used for RNA-Seq and qRT-PCR analyses.

The change of plant endogenous hormone and antioxidant enzyme activities in tomato plants feeding by *B. tabaci* was analyzed. JA titer was decreased and SA titer was increased in treatment group compared with the control (Figures 1B,C). And the difference was the greatest at 24 h. Then, we detected the antioxidant enzyme activities of tomato plants at this time point. Compared with the control, the activities of catalase (CAT), peroxidase (POD), and superoxide dismutase (SOD) were all increased (Figures 1D–F). To explore the molecular mechanisms of interaction between tomato plants and *B. tabaci*, samples from tomato plants feeding by *B. tabaci* 24 h were used for RNA-Seq.

### Overview of Tomato Plant Transcriptome Data

**FIGURE 2:**
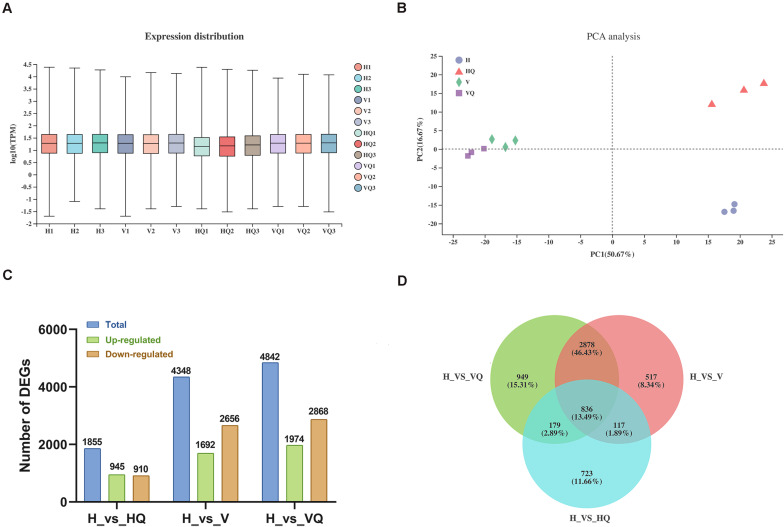
Global gene expression profiling and principal component analysis in tomato plants under different treatment. H, healthy tomato plants. HQ, tomato plants was fed on by healthy *B. tabaci* for 24 h. V, tomato plants was inoculated with ToCV. VQ, tomato plants with ToCV that were fed on by healthy *B. tabaci* for 24 h. **(A)** Density plot of global gene expression in tomato plants under different treatments. **(B)** The degree of similarity between tomato plants samples under different treatments. **(C)** Up- and down-regulated differentially expressed genes (DEGs). **(D)** Venn diagram of DEGs.

To reveal the molecular regulation of tomato plants under treatment with *B. tabaci* and ToCV, we performed high-throughput sequencing. Transcriptome analysis of 12 samples was completed, and a total of 86.56 Gb clean data was obtained (Supplementary Table 1). The clean data of each sample reached 6.29 Gb or more. The Q20 and Q30 base percentage was more than 98.76 and 95.83%, respectively. A total of 29,010 expressed genes were detected in this analysis, including 26,512 known and 2,498 new genes, as well as total of 45,887 expressed transcripts, including 25,484 known and 20,403 new transcripts. Compared with the control group, tomato plants transcript abundance changed significantly under the action of *B. tabaci* and ToCV (Figure 2A and Supplementary Table 2), and the correlation analysis between samples showed that there was a high correlation between biological replicates, indicating that the sequencing results were reliable (Figure 2B). Meanwhile, the expression of differential genes between the different treatment groups and the control group was counted (Figure 2C). A Venn diagram shows the number of common and unique DEGs between each group comparison (Figure 2D).

### Identification of Differentially Expressed Genes Under Different Treatments

**FIGURE 3:**
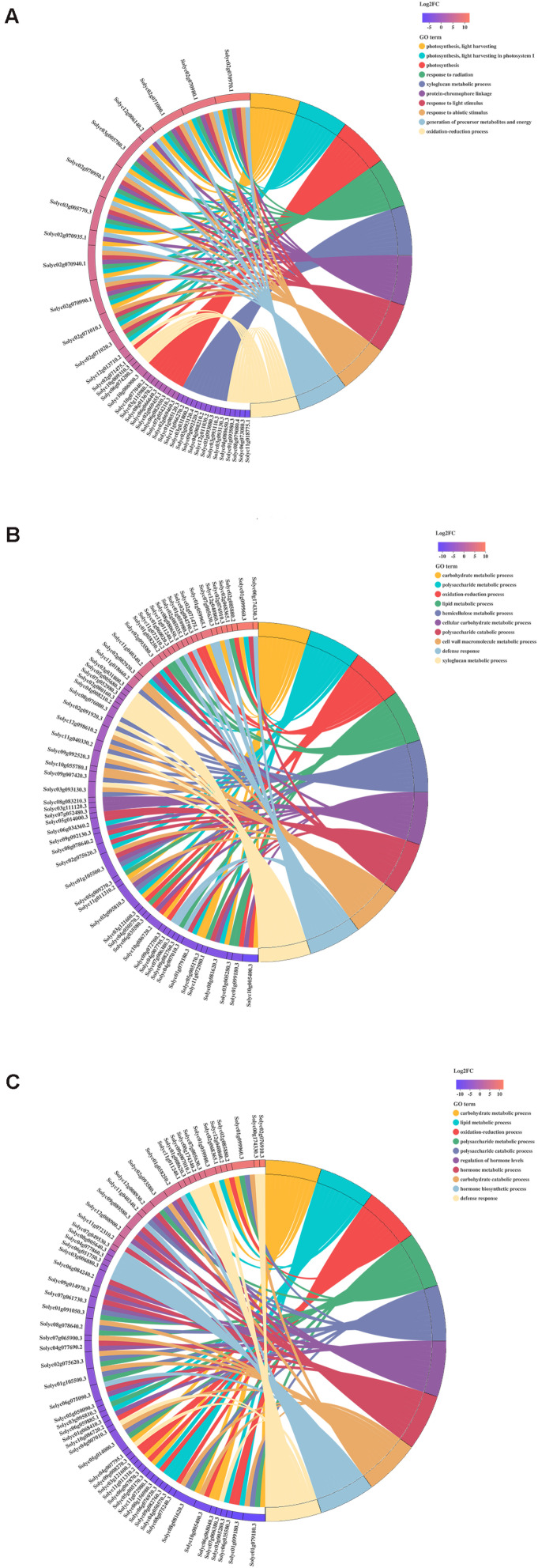
Chord plot of the top 10 Gene Ontology (GO) terms. **(A)** H_vs_HQ. **(B)** H_vs_V. **(C)** H_vs_VQ. Chords show a detailed relationship between the log_2_-fold change (log_2_FC) of differentially expressed genes (DEGs) (left semicircle) and their enriched GO terms (right semicircle). H, healthy tomato plants. HQ, tomato plants was fed on by healthy *B. tabaci* for 24 h. V, tomato plants was inoculated with ToCV. VQ, tomato plants with ToCV that were fed on by healthy *B. tabaci* for 24 h.

The Gene Ontology (GO) Chord plot shows the ten most enriched GO terms and top ten genes for each term. In H_vs_HQ, the most significant GO terms were light harvesting, light harvesting in photosystem I, photosynthesis, response to radiation, xyloglucan metabolic process, protein-chromophore linkage, response to light stimulus, response to abiotic stimulus, generation of precursor metabolites, and energy and oxidation-reduction process (Figure 3A). In H_vs_V, the most significant GO terms were carbohydrate metabolic process, polysaccharide metabolic process, oxidation-reduction process, lipid metabolic process, hemicellulose metabolic process, cellular carbohydrate metabolic process, polysaccharide catabolic process, cell wall macromolecule metabolic process, defense response and xyloglucan metabolic process (Figure 3B). In H_vs_VQ, the 10 most significant GO terms were carbohydrate metabolic process, lipid metabolic process, oxidation-reduction process, polysaccharide metabolic process, hemicellulose metabolic process, polysaccharide catabolic process, and regulation of hormone levels, hormone metabolic process, carbohydrate catabolic process, hormone biosynthetic process and defense response (Figure 3C).

**FIGURE 4:**
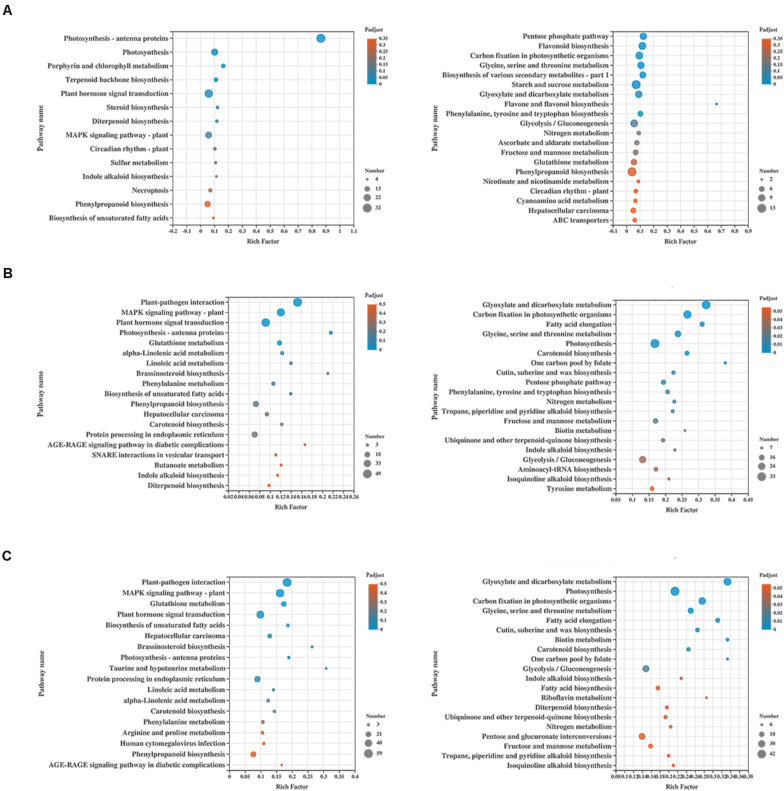
KEGG enrichment analysis of the differentially expressed genes (DEGs) in tomato plants. **(A)** KEGG enrichment analysis of H_vs_HQ. **(B)** KEGG enrichment analysis of H_vs_V. **(C)** KEGG enrichment analysis of H_vs_VQ. The left and right show the enrichment of up- and down-regulated genes, respectively. H, healthy tomato plants. HQ, tomato plants was fed on by healthy *B. tabaci* for 24 h. V, tomato plants was inoculated with ToCV. VQ, tomato plants with ToCV that were fed on by healthy *B. tabaci* for 24 h.

Kyoto Encyclopedia of Genes and Genomes (KEGG) enrichment was carried out to better understand the biological functions of common anti-stress genes in tomato plants. In H_vs_HQ, the up-regulated DEGs were mainly enriched in the pathway of photosynthesis - antenna proteins (Figure 4A), and its enrichment degree was much greater than other pathways. We found that the pathway includes many DEGs which encode chlorophyll a-b binding protein, and all of these genes were up-regulated (Supplementary Table 2). Previous study has proved the role of chlorophyll a-b binding protein in photosynthesis (Klimmek et al., 2006). This indicated that *B. tabaci* can induce enhanced photosynthesis in tomato plants. The down-regulated DEGs were mainly enriched in the pathway of pentose phosphate pathway and flavonoid biosynthesis (Figure 4A). However, in the two sets of data with the participation of ToCV, the up-regulated DEGs were mainly enriched in pathway of plant-pathogen interaction and MAPK signaling pathway - plant (Figures 4B,C). The down-regulated DEGs were mainly enriched in glyoxylate and dicarboxylate metabolism and carbon fixation in photosynthetic organisms (Figures 4B,C). This data indicated that ToCV induced high expression of tomato plants defense pathway-related genes.

**FIGURE 5:**
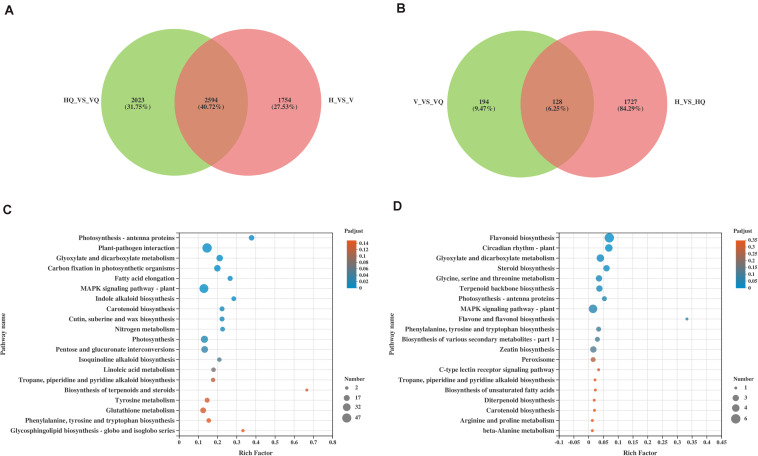
The effects of ToCV and *Bemisia tabaci* on tomatoes, respectively. **(A,B)** The Venn diagram of differentially expressed mRNAs. **(C)** KEGG enrichment analysis of the common differential genes in HQ_vs_VQ and H_vs_V. **(D)** KEGG enrichment analysis of the common differential genes in V_vs_VQ and H_vs_HQ. H, healthy tomato plants. HQ, tomato plants was fed on by healthy *B. tabaci* for 24 h. V, tomato plants was inoculated with ToCV. VQ, tomato plants with ToCV that were fed on by healthy *B. tabaci* for 24 h.

To get more information about how ToCV and *B. tabaci* affect gene expression in tomato plants, we increased comparison between HQ and VQ, V and VQ in the RNA-seq data. Then, DEGs from HQ_vs_VQ with those from H_vs_V, and DEGs from V_vs_VQ with those from H_vs_HQ were also compared (Figures 5A,B). We found that the common differential genes in HQ_vs_VQ and H_vs_V were mainly enriched in photosynthesis- antenna proteins, plant-pathogen interaction, glyoxylate and dicarboxylate metabolism (Figure 5C). The common differential genes in V_vs_VQ and H_vs_HQ were mainly enriched in flavonoid biosynthesis, circadian rhythm- plant, glyoxylate and dicarboxylate metabolism (Figure 5D). The comparison results showed that ToCV and *B. tabaci* infection can cause the differential expression of plant energy synthesis and photosynthesis-related genes, and that ToCV and *B. tabaci* can induce changes in plant-related anabolic metabolism. Meanwhile, we found there were more DEGs from H vs. VQ and H vs. V than H vs. HQ (Figure 2D). We speculate that this may be related to the complex resistance mechanism induced by virus infection. However, which differential gene plays a key role in the defense pathway requires further experiments.

### Analysis of DEGs Related to Plant Defense Response

Compared with healthy plants, the genes related to defense response in tomato plants were activated under different treatments. Among them, genes in typical disease resistance pathways, like plant-pathogen interaction and MAPK signaling pathway, were highly differentially expressed. For example, the expression of WRKY and CML genes can activate the defense response of plants, and differential expression of 9 WRKY (2 up- and 7 down-regulated), 17 WRKY (12 up- and 5 down-regulated), and 28 WRKY (20 up- and 8 down-regulated) genes was detected in HQ, V, and VQ, respectively (Supplementary Table 3). Seven CML (5 up- and 2 down-regulated), 6 CML (3 up- and 3 down-regulated), and 12 CML (9 up- and 3 down-regulated) genes was detected in HQ, V, and VQ, respectively (Supplementary Table 3). This indicates that the defense pathways of tomato plants were activated to fight *B. tabaci* and ToCV.

Notably, genes with IDs solyc05g055745.1 (down-regulated in HQ, V, and VQ) and solyc08g062490.3 (down-regulated in HQ and up-regulated in V and VQ) were detected to be differentially regulated in all comparisons (Supplementary Table 3). In the groups of tomato plants with ToCV infection, 16 DEGs overlapped between H_vs_V and H_vs_VQ. Among them, genes with IDs solyc08g062490.3 and solyc06g048870.2 were up-regulated more than ten-fold, indicating that they may play a crucial role in the pathway of tomato plants resistance to ToCV (Supplementary Table 3). Moreover, we noted many WRKY transcripts that have not been activated in HQ and V treatments were recorded in VQ. Among them, genes with IDs solyc05g015850.3 and solyc08g008273.1 showed the highest fold change of difference between up-regulated and down-regulated genes, respectively (Supplementary Table 3). These genes might have been activated only in the presence of a whitefly feeding on a virus-infected plant. Such transcripts could give more novel information related to virus-vector interaction.

### Analysis of DEGs Related to Photosynthesis

**FIGURE 6:**
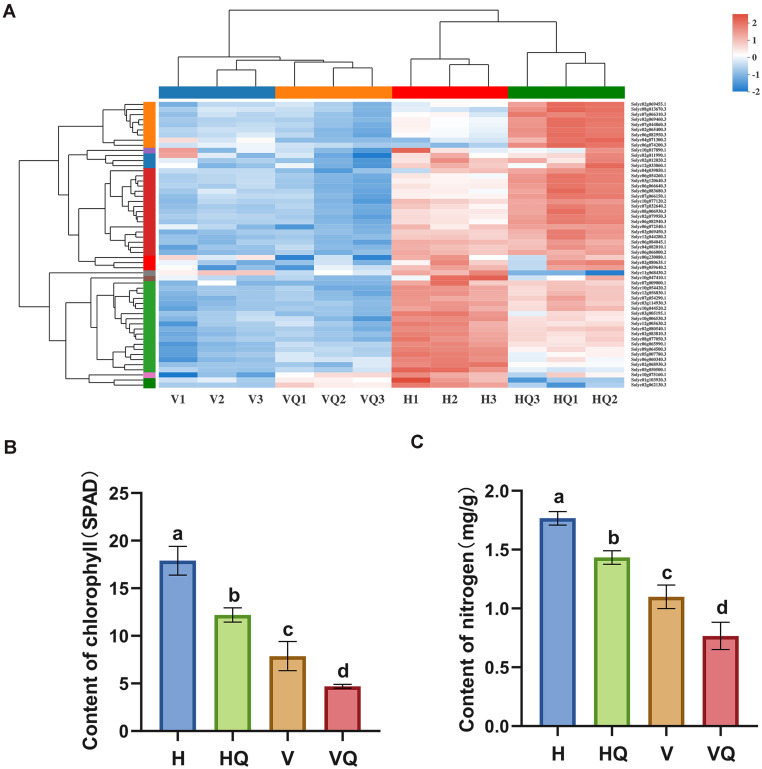
Effects of different treatments on photosynthesis of tomato plants. **(A)** Differentially expressed photosynthesis genes in tomato plants under different treatments. **(B)** Chlorophyll content of tomato plants under different treatments. **(C)** Nitrogen content of tomato plants leaves under different treatments. For each sample, means with different letters are significantly different (*P* < 0.05). H, healthy tomato plants. HQ, tomato plants was fed on by healthy *B. tabaci* for 24 h. V, tomato plants was inoculated with ToCV. VQ, tomato plants with ToCV that were fed on by healthy *B. tabaci* for 24 h.

A total of 56 DEGs related to photosynthesis were identified. Through cluster analysis of the expression patterns of DEGs, the results showed that the genes related to photosynthesis of tomato plants treated with the virus were significantly down-regulated (Figure 6A). This indicates that virus infection can strongly repress the photosynthetic pathway of tomato plants. At the same time, photosynthesis - antenna proteins were the most enriched pathway in our data. Among them, all DEGs were up-regulated in H_vs_HQ, while six genes were significantly down-regulated in H_vs_VQ and H_vs_V, respectively (Supplementary Table 4). The chlorophyll and nitrogen content of tomato leaves under different treatments were measured (Figures 6B,C), and the results showed that compared with healthy leaves, the content of both under each treatment group was reduced.

### Global Metabolic Pathways of the DEGs

**FIGURE 7:**
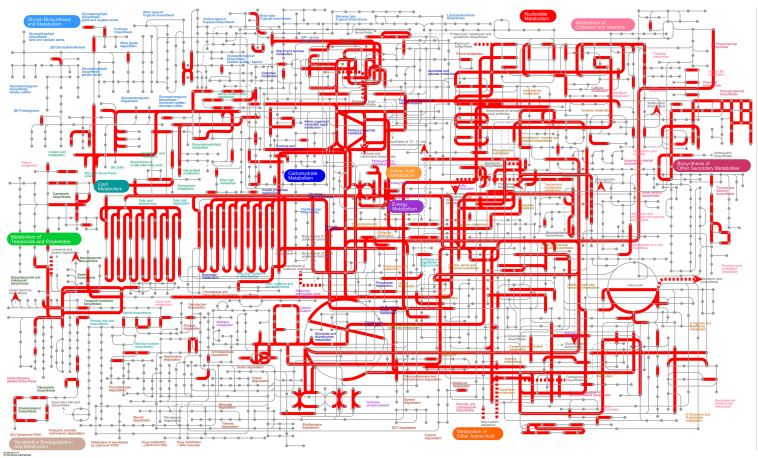
Overview of the metabolic pathways of DEGs between healthy tomato plants (H) and tomato plants under different treatments (V, VQ, and HQ). The red lines represent pathways annotated by DEGs. H, healthy tomato plants. HQ, tomato plants was fed on by healthy *B. tabaci* for 24 h. V, tomato plants was inoculated with ToCV. VQ, tomato plants with ToCV that were fed on by healthy *B. tabaci* for 24 h.

iPath3.0^1^ was used to visually analyze the metabolic pathways and view the metabolic pathway information of the entire biological system (Darzi et al., 2018). As shown in Figure 7, most DEGs were annotated to carbohydrate metabolism, energy metabolism, amino acid metabolism and lipid metabolism. Among them, the metabolic and synthetic pathways of various sugars were significantly enriched in carbohydrate metabolism, indicating that the energy metabolism of tomato plants is significantly affected under treatment with *B. tabaci* and ToCV.

### Global miRNA Expression Pattern

**FIGURE 8:**
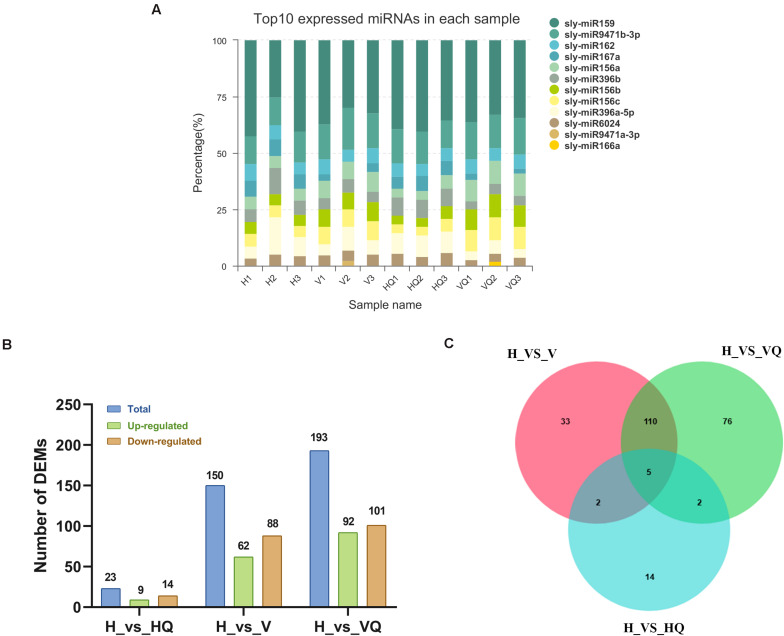
The expression profile of differentially expressed miRNAs at different treatment. **(A)** Top expressed miRNAs in each sample. **(B)** Up- and down-regulated differentially expressed miRNAs. **(C)** The Venn diagram of differentially expressed miRNAs. H, healthy tomato plants. HQ, tomato plants was fed on by healthy *B. tabaci* for 24 h. V, tomato plants was inoculated with ToCV. VQ, tomato plants with ToCV that were fed on by healthy *B. tabaci* for 24 h.

In total, 12 samples were sequenced and a total of 141.72 M raw reads were obtained. The use of statistical methods to calculate the base distribution and quality fluctuations of each cycle of all sequencing reads can intuitively reflect the sequencing quality of the sample and quality of library construction from a macroscopic perspective. Sequencing-related quality assessments were performed on the original sequencing data of each sample. The raw reads of each sample reached 10.27 M or more, and the Q30 base percentage was 89.98% or more. Then, the clean reads of each sample were aligned with the *Solanum lycopersicum* reference genome sequence (SL3.0), and the number of reads from the alignment ranged from 6556042 to 9737059 (Supplementary Table 5). The expression distribution map reflects the expression pattern of miRNA in each sample as a whole. Sly-miR159, sly-miR9471b-3p, and sly-miR162 were the most expressed miRNAs in each sample (Figure 8A and Supplementary Table 13).

The identification of differentially expressed miRNAs between the different treatment groups was performed on the basis of a *p*-value < 0.05 and | log2 (fold change)| > 1. By analyzing a volcano plot based on their expression, four differentially expressed clusters containing 242 miRNAs were identified. In H_vs_HQ, there were 9 up- and 14 down-regulated miRNAs (Figure 8B). In H_vs_V, the expression of 62 miRNAs was increased, and that of 88 miRNAs was decreased (Figure 8B), while in H_vs_VQ, there were 92 up- and 101 down-regulated miRNAs (Figure 8B). The Venn diagram shows the number of common and unique differentially expressed miRNAs between each group comparison (Figure 8C). This data suggested that tomato plants had different miRNA regulatory characteristics under different treatments with *B. tabaci* and ToCV and that miRNA changes very little when *B. tabaci* is feeding.

### Prediction and Functional Characterization of Potential Target mRNAs of miRNAs

The targets of these miRNAs were predicted using the online software psRobot. According to target gene prediction, 242 miRNAs had 1078 target genes. Then, GO and KEGG functional enrichments were performed to explore the distribution and potential biological functions of these 1078 candidate target genes (Supplementary Table 6).

For GO analysis, the target genes corresponding to the up-regulated miRNAs were enriched in the regulation of defense response to viruses and regulation of the immune effector process, while down-regulated target genes were enriched in ADP binding and UDP-*N*-acetylglucosamine biosynthetic process in H_vs_HQ (Supplementary Table 7). In H_vs_V, the target genes corresponding to up-regulated miRNAs were enriched in oligopeptide transport and peptide transport, while those corresponding to down-regulated miRNAs were enriched in ADP binding and chromatin binding (Supplementary Table 7). However, the target genes corresponding to up-regulated miRNAs were enriched in the lignin catabolic process and phenylpropanoid catabolic process in H_vs_VQ (Supplementary Table 7), and those corresponding to down-regulated miRNAs were enriched in chromatin binding and UDP-glucuronate decarboxylase activity (Supplementary Table 7).

For KEGG analysis, the targets of the up-regulated miRNAs in tomato plants under virus infection were enriched in pathways of various types of N-glycan biosynthesis, ABC transporters, and basal transcription factors, among others (Supplementary Table 8). The targets of the down-regulated miRNAs were enriched in the pathway of material metabolism process. Similarly, down-regulated miRNAs were enriched in the pathway of glucose metabolism in H_vs_VQ (Supplementary Table 8). The targets of up-regulated miRNAs were enriched in lysine degradation, arachidonic acid metabolism and pantothenate and CoA biosynthesis, among others (Supplementary Table 8). However, in H_vs_HQ, the targets of up-regulated miRNAs were enriched in DNA replication and necroptosis (Supplementary Table 8). The down-regulated miRNAs were enriched in purine metabolism, RNA polymerase and pyrimidine metabolism, among others (Supplementary Table 8).

### Conjoint Analysis of Small RNA-Seq and mRNA-Seq

**FIGURE 9:**
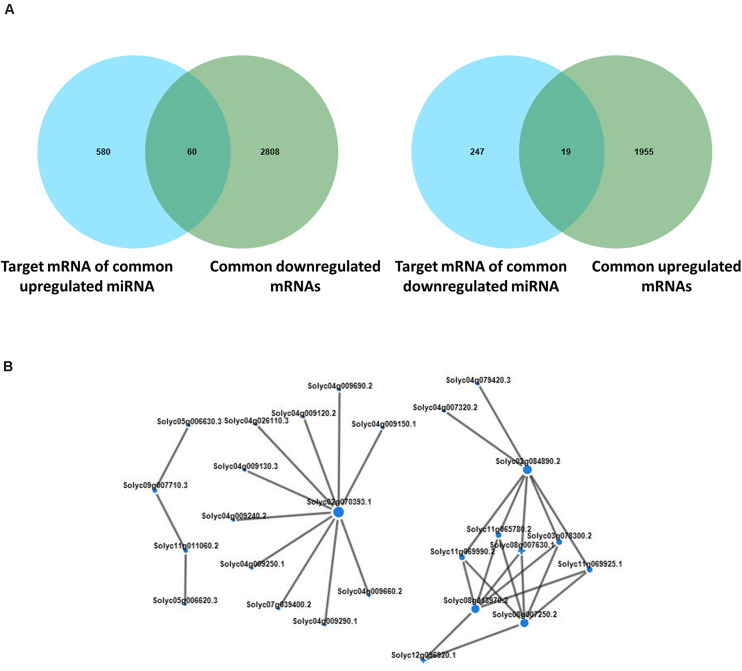
Conjoint analysis of differentially expressed genes and differentially expressed miRNAs. **(A)** Venn analysis of the predicted miRNA target mRNAs and differentially expressed mRNAs with the same expression trend in tomato plants under different treatment. The left panel shows the common highly expressed mRNAs, and the right panel shows the common lowly expressed mRNAs. **(B)** Interaction networks of the common up- and down-regulated miRNAs and target mRNAs. H, healthy tomato plants. HQ, tomato plants was fed on by healthy *B. tabaci* for 24 h. V, tomato plants was inoculated with ToCV. VQ, tomato plants with ToCV that were fed on by healthy *B. tabaci* for 24 h.

We conducted further analysis on the differential expression of miRNA and mRNA in tomato plants under co-treatment with *B. tabaci* and ToCV. In total, 60 down- and 19 up-regulated mRNAs were identified on the basis of a Venn map of the miRNA targets and common DEGs (Figure 9A). These common targets were conjointly analyzed in terms of enriched KEGG pathways and functionally enriched GO terms. The highly expressed target genes during both treatment with *B. tabaci* and ToCV were enriched in typical GO terms and pathways related to plant morphology development and amino acid metabolism process, such as the developmental process, simple leaf morphogenesis, leaf vascular tissue pattern formation, fatty acid degradation, alpha-Linolenic acid metabolism, and amino sugar and nucleotide sugar metabolism (Supplementary Tables 9, 10). In contrast, the lowly expressed target genes were enriched in typical GO terms and pathways related to metabolism, signal transduction and amino acid metabolism, such as lignin metabolic and catabolic process, phenylpropanoid metabolic and catabolic process, phosphatidylinositol signaling system, MAPK signaling pathway - yeast, and fatty acid elongation (Supplementary Tables 9, 10). Interaction networks of the common up- and down-regulated miRNAs and target mRNAs were constructed (Supplementary Table 11). The miRNA-mRNA pairs involved in disease resistance were selected and are shown in Figure 9B. Among them, genes solyc02g070393.1, solyc02g084890.2, solyc08g013970.2, and solyc08g007250.2 interacted with multiple target genes, indicating that they may play an important role in mediating plant disease resistance pathways.

### Validation of Sequencing Data by qRT-PCR

**FIGURE 10:**
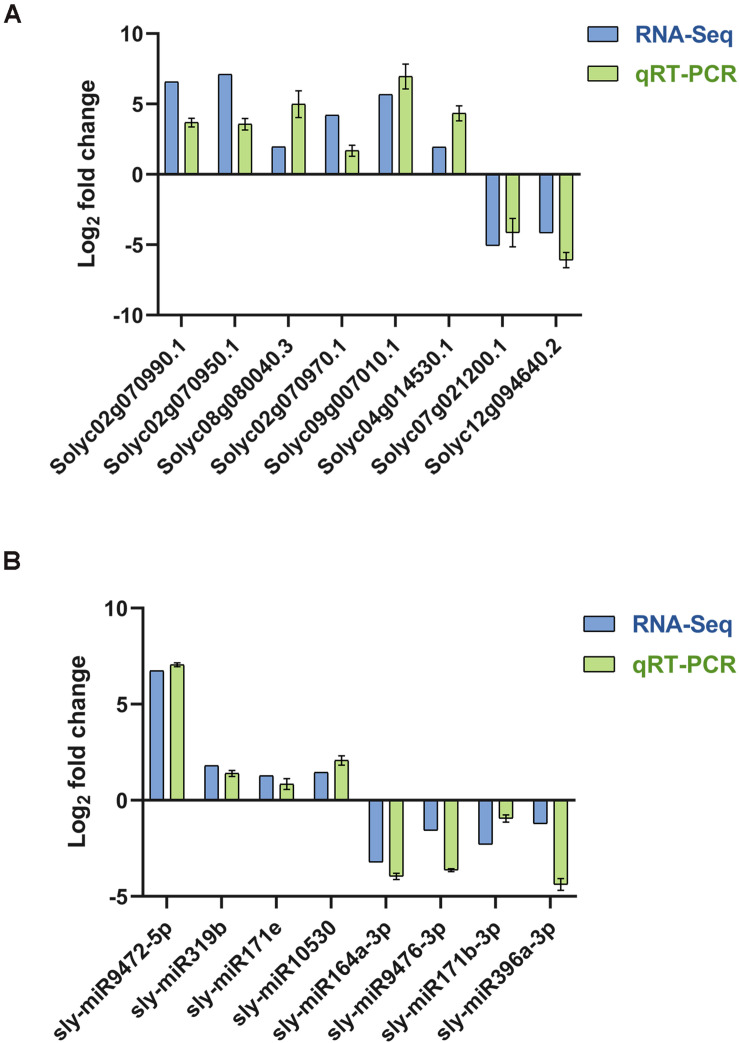
qRT-PCR verified the quality of transcriptome sequencing. **(A)** The relative expression levels of random mRNAs and the small RNA sequencing results. **(B)** The relative expression levels of random miRNAs and the small RNA sequencing results.

To verify the RNA-Seq data, 9 mRNAs and 8 miRNA were chosen for qRT-PCR. Primers in the primer sequence were used for qRT-PCR (Supplementary Table 12). The expression data in each of the selected genes obtained by qRT-PCR were consistent with the RNA-Seq results, indicating a similar trend between the transcriptome and qRT-PCR datasets (Figures 10A,B).

## Discussion

With the development of high-throughput sequencing technology, it has become widely used in the studies of plant-virus interactions (Ding et al., 2019; Şahin-Çevik et al., 2019; Fortes et al., 2020), and there have been a lot of plant-virus interactions in tomato plants, but most of them are concentrated on tomato yellow leaf curl virus (TYLCV) and related DNA viruses (Chen et al., 2013; Lucioli et al., 2016; Sahu et al., 2020). Although much research has focused on the identification of related resistance genes, it has always been a challenge to determine resistance genes so that they can be introduced into tomato plants. This study explored the molecular mechanism of tomato plants response to ToCV and *B. tabaci*. We identified genes related to ToCV and *B. tabaci* resistance in tomato plants, which could speed up the selection process of ToCV resistant tomato varieties.

Plants can reduce insect attacks by generating specific responses and activating different metabolic pathways, including chemical and physical barriers such as the induction of defensive proteins (Haruta et al., 2001; Mello and Silva-Filho, 2002). Previous studies demonstrated that *B. tabaci* can suppress JA defenses and thereby enhance *B. tabaci* performance (Su et al., 2018; Zhang et al., 2018). BtFer1, a salivary ferritin secreted into tomato plants during whitefly feeding, can reduces H_2_O_2_ levels, callose deposition, and proteinase inhibitors production, and represses JA-mediated defense responses (Su et al., 2019). Our data demonstrated that many genes about synthesis of protein-derived molecules, which involved in host-plant resistance to insects, including chitinases, proteinase inhibitors and peroxidase, were differentially regulated (Supplementary Table 2). Interestingly, compared with HQ_vs_H, more differentially expressed proteinase inhibitor genes were detected in VQ_vs_H (Supplementary Table 2). Among them, gene with ID solyc09g083445.1 was up-regulated in both comparisons, and up-regulated more than ten-fold changes with the action of ToCV. Moreover, we also found some proteinase inhibitor genes have been activated only in the presence of a *B. tabaci* feeding on a virus-infected plant, like solyc09g089505.1, and solyc09g089540.3 (Supplementary Table 2). Our results suggested that these genes which can help tomato plants reduce attack of *B. tabaci*, showed more complicated expression pattern with the participation of ToCV. But the complex regulation mechanism behind this requires further research.

Plants build the first line of defense through pattern PRRs that recognize pathogens, and the resistance response of plants is an extremely sophisticated and complex cascade process (Jagodzik et al., 2018). These receptors can recognize ToCV and induce resistance responses (Shiu and Bleecker, 2001; Beck et al., 2012). Members of WRKY Group III transcription factors are important in TYLCV defense signaling pathway in tomato (Huang et al., 2016). Our results showed some WRKY genes that have been significantly regulated in the presence of ToCV and *B. tabaci* in tomato plants (Supplementary Table 3). These results suggest that downstream PR genes were activated by these WRKY genes and induced resistance of tomato plants to ToCV. Previous research has showed that the binding of Ca^2+^ to CML can promote the autoimmune response of plants (Ma et al., 2008). Only one gene (solyc03g115930.2) was detected to be differentially regulated in all comparisons, and it was down-regulated in H_vs_HQ, while up-regulated in H_vs_V and H_vs_VQ (Supplementary Table 3). The gene with ID solyc06g069740.1 was up-regulated to the maximum fold change in the virus treatment group compared with healthy tomato plants (Supplementary Table 3). However, in the group of tomato plants without ToCV infection, the gene was not detected to be differentially expressed. This may be mean that the gene play an important role in the way of promote the autoimmune response of tomato plants to ToCV. In summary, virus-infected tomato plants can activate pattern recognition receptors and promote the expression of downstream WRKY and CML, inducing plant defense responses.

The hypersensitive response (HR) is a form of programmed cell death (PCD) of plants to resist pathogen invasion, it is usually induced at the site of infection or a specific area around the site of infection and restricts the growth of pathogens (Dickman et al., 2001; Kim et al., 2008). The molecular chaperone complex formed by the SUPPRESSOR OF THE G2 ALLELE OF SKP1 (SGT1), heat shock protein 90 (HSP90), and a cysteine- and histidine-rich domains (CHORD)-containing protein that is an important signaling component of plant immune responses (Zhang et al., 2010). These genes were significant differentially regulated in our results. However, SGT1 only was up-regulated in the H_vs_VQ, but wasn’t differentially expressed in the H_vs_V (Supplementary Table 3). The enhancement of HR induced by Rp1-D21 associated with suppression of the *hydroxycinnamoyltransferase* (HCT) and *caffeoyl-CoA O-methyltransferase* (CCoAOMT) genes in maize (Murphree et al., 2020). The consistent results also found in our data (Supplementary Table 3). Moreover, many disease resistance protein which contain nucleotide binding site and C-terminal Leucine-rich repeat domains (NB-LRR) class was significantly regulated from our data, and most of them have higher up-regulation multiples in H_vs_VQ than in H_vs_V (Supplementary Table 3). This may be related to the participation of *B. tabaci*. The regulation mechanism behind this phenomenon is worthy of our further study.

According to Lei et al. (2017), leaf chlorosis induced by plant virus infection has a short fluorescence lifetime, which reflects damaged photosynthetic complexes and degraded chloroplasts (Lei et al., 2017). Tomato plants infected with ToCV showed chlorosis and dwarf. This may be that ToCV infection of tomato plants significantly affected the photosynthesis pathway of plants, leading to insufficient photosynthesis and the inability to produce energy needed for growth. Interestingly, we also observed that photosynthesis-related pathways and GO terms were significantly enhanced in H_vs_HQ, while related genes were down-regulated under the action of ToCV (Figure 4A and Supplementary Table 4). We speculate that plants increase their own defense by enhancing their photosynthesis, while viruses can inhibit photosynthesis-related genes expression in tomato plants. In addition, pathways related to sugar metabolism were significantly down-regulated, which was also an important reason why plant growth was affected (Figures 4B,C). Therefore, tomato plants infected by ToCV were usually dwarf and small, which was consistent with previous studies (Farooq et al., 2019; Zhao et al., 2020). We observed that photosynthesis-related genes were differentially regulated under different treatments (Figure 6A). Then, we measured the chlorophyll and nitrogen content of four groups of tomato plant samples. The data showed that compared with the control, the tomato plants under the other three treatments showed a gradual decline, and the tomato plants infected with ToCV were lower than the tomato plants with only *B. tabaci* (Figures 6B,C). A previous study revealed that the light-harvesting chlorophyll a/b-binding (Lhc) proteins function in multiple processes that are critical to plant growth, development, and abiotic stress response (Luo et al., 2019). Our data showed that Lhc-related genes were all up-regulated in H_vs_HQ, while some genes were down-regulated in tomato plants with ToCV (Supplementary Table 4). Among them, solyc06g069730.3, solyc08g067320.2, and solyc07g022900.3 were greatly changed under treatments with and without ToCV. This suggests that these three genes may play an important role in capturing pigments in tomato plants after ToCV infection.

Previous studies have analyzed the role of miRNA in the interaction between tomato plants and plant pathogens, such as tomato-ToLCNDV17 (Pradhan et al., 2015), tomato-*Botrytis cinerea* (Jin and Wu, 2015), and tomato-*Fusarium* (Ouyang et al., 2014). These studies pointed out that miRNAs were involved in plant defense pathways. However, there are few studies on the joint analysis of miRNA and mRNA expression profiles of the interaction between tomato virus and vectors. Hence, a comprehensive study of the miRNA and mRNA expression profiles and correlating miRNA-mRNA expression in tomato plants under the effect of *B. tabaci* and ToCV is well timed. Through the analysis of our data, the top ten miRNAs with the highest expression levels in each sample were determined. Most of their targeted gene descriptions were related to disease resistance pathways, such as disease resistance protein, NBS-LRR resistance protein-like protein, and R2R3MYB transcription factor (Supplementary Table 6). These changes indicated that miRNAs mediated defense pathways in tomato plants and were significantly affected by viruses and *B. tabaci*.

Studies have confirmed the important role of specific miRNAs for plants against different plant viruses and pathogens (Zhang et al., 2016). MiR156 has been proven to regulate the initial growth and development of plants and resist abiotic stress (Zheng et al., 2019). Also in our study, the expression of miR156 was affected, verifying this result (Supplementary Table 6). A close inspection of the miRNAs regulated as an effect of *Alternaria solani* (Sarkar et al., 2017) and our study in tomato plants found that 4 miRNAs were commonly regulated by both stresses in tomato plants. Among them, sly-miR6022 and sly-miR171e were up-regulated; however, sly-miR396a-3p and sly-miR166c-5p were down-regulated. These 4 miRNAs showed a similar trend of regulation among the two stresses (Supplementary Table 13). In order to explore the differences in tomato response to early blight and ToCV, we compared the results of two studies. We found the DEGs in tomato response to early blight disease, were most significant related to pathways of response to stimulus process, photosynthesis, biosynthesis of secondary metabolites, plant-pathogen interaction and plant hormone signal transduction (Sarkar et al., 2017). This indicates that tomato response to ToCV and early blight has similarities. A comparison of the regulated miRNAs between biotrophic pathogen CMV (Feng J. et al., 2014) and ToCV stress in tomato plants showed that they do not have the same differential miRNAs. We found that the number of differential miRNAs in tomato plants under the action of *B. tabaci* was much lower than that under the action of ToCV (Figure 8C). Interestingly, sly-miR171f, sly-miR164b-5p, sly-miR164a-5p, and sly-miR171a were the most down-regulated under treatment with ToCV (Supplementary Table 13). This suggested that they may play an important role in mediating plant defense pathways to ToCV of tomato plants. In addition to known miRNAs, we also found 160 differentially expressed novel putative miRNAs (Supplementary Table 14); however, whether these genes have real biological activity requires further experimental verification.

miRNA functions by post-transcriptional regulation of its target genes, and the expression level of miRNA is inversely proportional to mRNA (Zhao et al., 2018). We used the combined analysis of miRNA and mRNA in the transcriptome range to explore the miRNA-mRNA pairs regulated in the tomato-ToCV-*B. tabaci* interaction process. In our data, functional annotation analysis showed that these potential targets were mainly involved in disease response and metabolic processes (Supplementary Tables 9, 10). The miRNA-mRNA pairs involved in disease resistance were selected and are shown in Figure 8B, and we think genes solyc02g070393.1, solyc02g084890.2, solyc08g013970.2, and solyc08g007250.2 may play an important role in mediating plant disease resistance pathways. This requires us to verify them to reach a conclusion. Overall, these data strongly support the hypothesis that miRNAs play important roles in the regulation of immune responses against *B. tabaci* and ToCV in tomato plants.

## Materials and Methods

### Tomato Plants Materials and Sample Preparation

Tomato seeds (*S. lycopersicum* Mill. Cv. Zuanhongmeina, no resistance to ToCV and *B. tabaci*) were sown in a plastic seedling tray (53 × 27.5 × 4.5 cm) with a nutrient substrate and planted in an insect-proof cage in the greenhouse (26 ± 1°C, RH70% ± 5%, Photoperiod L//D = 16 h//8 h) on the sixth floor of the Hunan Institute of Plant Protection, without contact with any pesticides or insects. The seedlings were transplanted into pots with a diameter of 10 cm until they had 2–3 true leaves. The *B. tabaci* used in this experiment were *B. tabaci* MED, which was presented by Dr. Youjun Zhang’s research group from the Institute of Vegetables and Flowers, Chinese Academy of Agricultural Sciences. All of the *B. tabaci* used in our study were virus-free.

The infectious cDNA clone of ToCV was provided by Prof. Tao Zhou (China Agricultural University). The preparation of ToCV-infected tomato plants were performed as described (Zhao et al., 2016). First, plasmids containing RNA1 (pCa-ToCR1) and RNA2 (pCa-ToCR2) were placed respectively in two liquid YEP mediums containing 50 mg/mL kanamycin and 50 mg/mL rifampicin. After overnight cultured at 28°C, the bacterium containing ToCV-RNA1 and ToCV-RNA2 were obtained. After the bacterial solutions were collected and centrifuged, the prepared suspension (includes 10 mM MES, 10 mM MgCl_2_, and 200 mM *Acetosyringone*) was used to resuspend the precipitated bacterium, and further diluted to OD600 = 1.0. The *Agrobacterium* suspension of pCa-ToCR1 and pCa-ToCR2 were mixed at a volume ratio of 1:1. Then, 0.5 mL ToCV infectious cDNA agro clone was injected into the three-true-leaf stage tomato plants. Both visual (leaf chlorosis) and molecular (RT-PCR) inspections were conducted to confirm the viral infection (Shi et al., 2018). Due to the low inoculation efficiency (about 10%), a large number of repetitions were done in order to obtain enough ToCV-infected tomato plants.

The tomato plants were divided into four groups. The first group was a control, which grew 40 days normally. In the second group, every tomato plant (40 days old) was foraged by 300 *B. tabaci* adults (virus-free) for 24 h. The third group of tomato plants was inoculated with ToCV for 40 days. In the fourth group, every tomato plant infected with ToCV for 40 days and it were foraged by 300 *B. tabaci* adults (virus-free) for 24 h. After plants were foraged by *B. tabaci* adults for 24 h, all the whiteflies were removed by airflow in the aspirating equipment, in this way leaf tissue was not touched and damaged. Meanwhile, the forged tomato leaves were sampled, and the plants in the first and third group were also sampled. The four group of samples were named H, HQ, V and VQ. There were six tomato plants in each treatment, with three biological replicates. A total of 12 samples of tomato leaves were used for subsequent experiments and analysis. Detailed sequence information of ToCV-specific RT-qPCR primers is given in Supplementary Table 12.

### Quantification of Plant Endogenous Hormone and Analysis of Antioxidant Enzyme Activities

The 40-day-old tomato plants infested by virus-free *B. tabaci* and without *B. tabaci* feeding were used for quantification of plant endogenous hormone with 1 g/plant. After feeding times of 0, 12, 24, 36, and 72 h, the clip cages and whiteflies within were removed, and the corresponding leaves were collected. The entire plant received the same treatment, and each treatment was represented by three replicates. Leaves of tomato were ground with 10 mL isopropanol/hydrochloric acid and shaken at 4°C for 30 min (You et al., 2016). Then, 20 mL dichloromethane was added. The mixture was shaken at 4°C for 30 min and centrifuged at 13,000 rpm, 4°C for 5 min. The organic fraction was separated and then dried under nitrogen in darkness. The solid residue was re-suspended in 400 μL methanol/0.1% methanoic acid. Sample was filtered with a 0.22 μm filter membrane before HPLC-MS/MS analysis. HPLC analysis was performed using a poroshell 120 SB-C18 (Agilent, United States) column (150 mm × 2.1 mm × 2.7 μm). The mobile phase A solvents consisted of methanol + 0.1 % methanoic acid and the mobile phase B solvents consisted of ultrapure water + 0.1 % methanoic acid. The injection volume was 2 μL. MS conditions were as follows: the spray voltage was 4500 V; the pressure of the air curtain, nebulizer, and aux gas were 15, 65, and 70 psi, respectively; and the atomizing temperature was 400°C. Antioxidant enzyme activity is detected by catalase activity detection kit (Solarbio, Beijing, China), peroxidase activity detection kit (Solarbio, Beijing, China), and SOD activity detection kit (Solarbio, Beijing, China) according to the manufacturer’s instructions, respectively.

### RNA Extraction, Library Preparation, and Sequencing

Total RNA was extracted from 12 samples of tomato leaves using TRIzol^®^ Up according to the manufacturer’s instructions (Invitrogen, Carlsbad, CA, United States). Genomic DNA was removed using DNase I RNase-free (TaKara, Beijing, China). RNA degradation and contamination were monitored on 1% agarose gels. The RNA concentration was measured using a ND-2000 (Thermo Fisher Scientific, Beijing, China). Finally, RNA integrity was assessed using a 2100 Bioanalyzer (Agilent Technologies, Santa Clara, CA, United States). The RNA-seq transcriptome library was prepared following the TruSeq^TM^ RNA sample preparation kit from Illumina (San Diego, CA, United States) using 1 μg of total RNA. Shortly, mRNA was isolated according to the polyA selection method by oligo(dT) beads and then fragmented by fragmentation buffer. Double-stranded cDNA was synthesized using a SuperScript double-stranded cDNA synthesis kit (Invitrogen, Carlsbad, CA, United States) with random hexamer primers (Illumina). Then, the synthesized cDNA was subjected to end-repair, phosphorylation and “A” base addition according to Illumina’s library construction protocol. Libraries were size selected for cDNA target fragments of 300 bp on 2% low range ultra-agarose followed by PCR amplification using Phusion DNA polymerase (NEB) for 15 PCR cycles. After being quantified by TBS380, the paired-end RNA-seq sequencing library was sequenced with the Illumina HiSeq X ten/NovaSeq 6000 sequencer (2 × 150 bp read length).

### Library Preparation for Small RNA Sequencing

A total amount of 3 μg total RNA per sample from 12 samples of tomato leaf was used as input material for the small RNA library. Sequencing libraries were generated using the NEBNext^®^ Multiplex Small RNA Library Prep Set for Illumina^®^ (NEB, United States) following the manufacturer’s recommendations and index codes were added to attribute sequences to each sample. The NEB 3′ SR adaptor was directly and specifically ligated to 3′ end of miRNA, siRNA and piRNA. After the 3′ ligation reaction, the SR RT primer was hybridized to the excess of the 3′ SR adaptor, which remained free after the 3′ ligation reaction and transformed the single-stranded DNA adaptor into a double-stranded DNA molecule. This step was important to prevent adaptor-dimer formation besides, dsDNAs were not substrates for ligation mediated by T4 RNA ligase 1 and, therefore, did not ligate to the 5′ SR adaptor in the subsequent ligation step. The 5′end adapter was ligated to the 5′end of miRNAs, siRNAs and piRNAs. Then, first-strand cDNA was synthesized using M-MuLV Reverse Transcriptase (RNase H–). PCR amplification was performed using LongAmp Taq 2× Master Mix, SR Primer for Illumina and index (X) primer. PCR products were purified on an 8% polyacrylamide gel (100 V, 80 min). DNA fragments corresponding to 140–160 bp (the length of small non-coding RNA, plus the 3′ and 5′ adaptors) were recovered and dissolved in 8 μL elution buffer. Library quality was assessed on the Agilent Bioanalyzer 2100 system using DNA High Sensitivity Chips. After cluster generation, the library preparations were sequenced on an Illumina Hiseq 2500/2000 platform and 75 bp single-end reads were generated.

### Read Mapping

The raw reads were trimmed and quality controlled by SeqPrep and Sickle with default parameters. Then clean reads were separately aligned to the *S. lycopersicum* reference genome sequence (SL3.0) with orientation mode using HISAT2 (Kim et al., 2015) software. The mapped reads of each sample were assembled by StringTie in a reference-based approach (Pertea et al., 2015).

### Differential Expression Analysis and Functional Enrichment

To identify DEGs between two different samples, the expression level of each transcript was calculated according to the transcripts per million reads (TPM) method. RSEM (Li and Dewey, 2011) was used to quantify gene abundances. Essentially, differential expression analysis was performed using DESeq2 (Love et al., 2014)/DEGseq (Wang et al., 2010), and EdgeR (Robinson et al., 2010) with a *Q* value ≤ 0.05. DEGs with | log2FC| > 1 and *Q* value ≤ 0.05 (DESeq2) were considered to be significant DEGs. Significant differently expressed miRNAs were extracted with | log2FC| > 1 and FDR < 0.05 by DEseq2. In addition, functional-enrichment analysis, including GO and KEGG were performed to identify which DEGs were significantly enriched in GO terms and metabolic pathways at the Bonferroni-corrected *P*-value ≤ 0.05 compared with the whole-transcriptome background. GO functional enrichment and KEGG pathway analysis were carried out by Goatools and KOBAS (Xie et al., 2011).

### Integrated Analysis of miRNA and mRNA Expression Profiles

The miRNA-mRNA pairs were determined by the target gene prediction results of miRNAs and biological function analyses of DEGs. Then, the networks of miRNA and mRNA expression profiles were displayed using networkX under Python after obtaining the protein interaction network relationship through the STRING database.

### Determination of Chlorophyll and Nitrogen Content

The OK-Y104 chlorophyll meter (Oukeqi, China) was used to measure tomato leaves under different treatments and control groups. Each tomato plant was measured three times on the upper, middle and lower leaves of each plant to prevent measurement error.

### qRT-PCR Validation

To verify the results of RNA-Seq, DEGs were randomly selected for qRT-PCR verification. Total RNA extraction and genomic DNA removal were performed as described above. The first-strand cDNA of mRNA was synthesized from 1 μg of RNA using a HiScript^®^ 1st Strand cDNA Synthesis kit (Vazyme, China). First-strand cDNA of miRNA was synthesized using miRNA 1st strand cDNA synthesis kit (by stem-loop) (Vazyme, China) according to the manufacturer’s instructions. qRT-PCR was carried out using a LightCycler^®^ 96 Real-Time PCR System (Roche, Basel, Switzerland), and the relative expression levels of target genes were calculated using the 2^–ΔΔCt^ method. Tomato genes Actin and UBI were selected as the housekeeping genes (Supplementary Table 12).

### Data Analysis

IBM SPSS Statistics 21 (SPSS Inc., Chicago, IL, United States) was used for statistical analysis. One-way ANOVA was conducted to compare accumulation of ToCV in tomato plants at different time points, chlorophyll content and nitrogen content of tomato plants leaves under different treatments.

## Data Availability Statement

The RNA-seq reads have been submitted to the SRA at NCBI under the accession PRJNA699095.

## Author Contributions

X-BS and YL conceived and designed the experiments. HY and L-PH performed the experiments. HY analyzed the data. D-Y-HL, Z-HZ, ZZ, D-YZ, X-GZ, L-MZ, YG, and X-QT contributed reagents, materials, and analysis tools. HY and X-BS wrote the manuscript. All authors contributed to the article and approved the submitted version.

## Conflict of Interest

The authors declare that the research was conducted in the absence of any commercial or financial relationships that could be construed as a potential conflict of interest.

## Footnotes

1 http://pathways.embl.de

## Supplementary Material

Supplementary Table 1 Quality of the transcriptome data of each sample.

Supplementary Table 2 Differentially expressed mRNAs between the different treatment groups.

Supplementary Table 3 Differentially regulated genes related to plant defense response.

Supplementary Table 4 Differentially expressed photosynthesis - antenna proteins in tomato plants under different treatments.

Supplementary Table 5 Quality control data statistics.

Supplementary Table 6 DEMs and their target genes.

Supplementary Table 7 GO enrichment analysis of target genes.

Supplementary Table 8 KEGG enrichment analysis of target genes.

Supplementary Table 9 GO enrichment analysis of the common targets.

Supplementary Table 10 KEGG enrichment analysis of the common targets.

Supplementary Table 11 Common up- and down-regulated miRNAs and target mRNAs.

Supplementary Table 12 Primers use in this study.

Supplementary Table 13 Differentially expressed miRNAs between the different treatment groups.

Supplementary Table 14 Novel putative miRNAs.
